# Application of nanopore adaptive sequencing in pathogen detection of a patient with *Chlamydia psittaci* infection

**DOI:** 10.3389/fcimb.2023.1064317

**Published:** 2023-01-23

**Authors:** Yanfeng Lin, Yan Dai, Shuang Zhang, Hao Guo, Lang Yang, Jinhui Li, Kaiying Wang, Ming Ni, Zongqian Hu, Leili Jia, Huiying Liu, Peng Li, Hongbin Song

**Affiliations:** ^1^ Academy of Military Medical Sciences, Academy of Military Sciences, Beijing, China; ^2^ Chinese PLA Center for Disease Control and Prevention, Beijing, China; ^3^ State Key Laboratory of Translational Medicine and Innovative Drug Development, Jiangsu Simcere Diagnostics Co., Ltd., Nanjing, China; ^4^ Institute of Health Service and Transfusion Medicine, Beijing, China; ^5^ Beijing Institute of Radiation Medicine, Beijing, China; ^6^ College of Pulmonary & Critical Care Medicine, 8th Medical Center, Chinese PLA General Hospital, Beijing, China

**Keywords:** nanopore adaptive sequencing, mNGS, pathogen detection, respiratory infection, *Chlamydia psittaci*

## Abstract

**Introduction:**

Nanopore sequencing has been widely used in clinical metagenomic sequencing for pathogen detection with high portability and real-time sequencing. Oxford Nanopore Technologies has recently launched an adaptive sequencing function, which can enrich on-target reads through real-time alignment and eject uninteresting reads by reversing the voltage across the nanopore. Here we evaluated the utility of adaptive sequencing in clinical pathogen detection.

**Methods:**

Nanopore adaptive sequencing and standard sequencing was performed on a same flow cell with a bronchoalveolar lavage fluid sample from a patient with *Chlamydia* psittacosis infection, and was compared with the previous mNGS results.

**Results:**

Nanopore adaptive sequencing identified 648 on-target stop receiving reads with the longest median read length(688bp), which account for 72.4% of all *Chlamydia* psittaci reads and 0.03% of total reads in enriched group. The read proportion matched to *C. psittaci* in the stop receiving group was 99.85%, which was much higher than that of the unblock (<0.01%) and fail to adapt (0.02%) groups. Nanopore adaptive sequencing generated similar data yield of *C. psittaci* compared with standard nanopore sequencing. The proportion of *C. psittaci* reads in adaptive sequencing is close to that of standard nanopore sequencing and mNGS, but generated lower genome coverage than mNGS.

**Discussion:**

Nanopore adaptive sequencing can effectively identify target *C. psittaci* reads in real-time, but how to increase the targeted data of pathogens still needs to be further evaluated.

## Introduction

Rapid and accurate pathogen detection is a prerequisite for treatment of infectious disease. Pathogen isolation and culture is the “gold standard” for the diagnosis of clinical infectious diseases, but it has a low positive rate and a long culture period, which is not conducive to rapid clinical diagnosis (Fenollar and Raoult, 2007). PCR-based nucleic acid detection is one of the most widely used pathogen detection methods at present, which has the advantages of short turnaround time, high specificity and sensitivity, simple operation process, and relatively low cost (Deng et al., 2020). However, it requires *a priori* knowledge of targeted pathogens and is unable to detect unknown pathogens.

Pathogen detection based on metagenomic sequencing can achieve unbiased identification of pathogens in clinical samples. Compared with traditional next-generation sequencing (NGS), nanopore sequencing has the advantages of real-time, long read length, portability, etc., and has been widely used in pathogen detection of clinical infectious diseases (Gu et al., 2021; Jabeen et al., 2022; Street et al., 2022). Limited by the high host background in the clinical samples and low data yield of nanopore sequencing, it is critical to achieve the enrichment of target pathogens for nanopore metagenomic sequencing. Several methods have been developed for pathogen enrichment such as multiplex PCR amplification (Quick et al., 2017), bait capture methods (Schuele et al., 2020) and saporin-based host DNA depletion (Charalampous et al., 2019), but they require additional laboratory processes and extend the turnaround time, and were only suitable for specific pathogens.

Recently, Oxford Nanopore Technologies has launched adaptive sequencing function, which can perform real-time basecalling and sequence alignment with a given reference genome during the read was sequenced. The voltage across the nanopore can be reversed to eject the unwanted sequences, so as to achieve the enrichment of the target sequences (Loose et al., 2016). The enrichment method based on adaptive sequencing does not require complicated steps and extra time during sample processing, and the enrichment of target sequences can be achieved only at the sequencing level. Previous studies have applied adaptive sequencing in target gene enrichment and variant detection of human genomes (Miller et al., 2021; Mariya et al., 2022; Stevanovski et al., 2022), specific species enrichment in simulated microbial communities (Payne et al., 2021; Kovaka et al., 2021; Martin et al., 2022)and microbiome profiling of human or animal samples (Marquet et al., 2022; Ong et al., 2022a; Ong et al., 2022b). Recently, adaptive sequencing has been successfully applied for the identification and enrichment of bacterial or viral pathogens in respiratory samples (Gan et al., 2021; Cheng et al., 2022; Lin et al., 2022). However, the enrichment effect of adaptive sequencing for targeted pathogen sequences in clinical samples remains to be further studied.

In this study, nanopore adaptive sequencing was performed on the bronchoalveolar lavage fluid sample from a patient with *Chlamydia psittaci* infection to evaluate the utility of nanopore adaptive sequencing for pathogen detection of clinical samples.

## Materials and methods

### Sample collection and nucleic acid extraction

The bronchoalveolar lavage fluid sample was collected from a 60-year-old female patient who was admitted to the hospital for “intermittent fever, dry cough, and fatigue”. The metagenomic next-generation sequencing (mNGS) of the bronchoalveolar lavage fluid sample was performed in previous study (Wang et al., 2021). Nucleic acid was extracted from the remaining bronchoalveolar lavage fluid sample using the QIAamp MinElute Virus Spin Kit (Qiagen, Hilden, Germany) according to the kit instructions, and the DNA concentration was quantified using a Qubit 3.0 Fluorometer (Thermo Fisher Scientific, CA, USA).

### Library preparation and nanopore adaptive sequencing

200ng of DNA was used for library preparation with ligation sequencing kit SQK-LSK109 (Oxford Nanopore Technologies, Cambridge, UK) according to the manufacturer’s instructions, and 0.8×AMPure XP Bead (Beckman Coulter, Indianapolis, United States) was used for the clean-up of the library after adding adapters. The final library concentration was quantified by Qubit 3.0 Fluorometer, and 75ng of the library was loaded into a FLO-MIN106 R9.4 flow cell and sequenced on a GridION platform with MinKNOW software (v21.05.12). Adaptive sequencing was performed in enrichment mode and the whole genome sequence of *C. psittaci* strain L99 (GenBank accession number: JACAAQ000000000) (Wang et al., 2021) obtained in the previous study was selected as the reference sequence. Half of the channels (1-256) in the flow cell were set as enriched group (perform adaptive sequencing) and the remaining channels (257-512) were set as control group (perform standard sequencing).

### Bioinformatic analysis

According to the channel number of each read, the sequencing data was divided into enriched group and control group. Based on the adaptive sequencing log file generated by MinKNOW, the reads corresponding to the enriched group were further divided into three groups according to the decisions of adaptive sequencing: stop receiving (the accepted target reads), unblock (the rejected non-target reads) and fail to adapt (reads could not be classified as accept or reject). The read length distributions of different groups were visualized using a box plot. Using *C. psittaci* strain L99 as the reference genome, the sequenced reads were aligned and indexed using minimap2 (v2.21) (Li, 2018) and SAMtools (v1.13) (Li et al., 2009). The number of reads, bases, genome coverage, and genome sequencing depth of *C. psittaci* in different groups were calculated every 15 minutes, and compared with the previous mNGS results.

## Results

### Overall performance of nanopore adaptive sequencing

**Figure 1:**
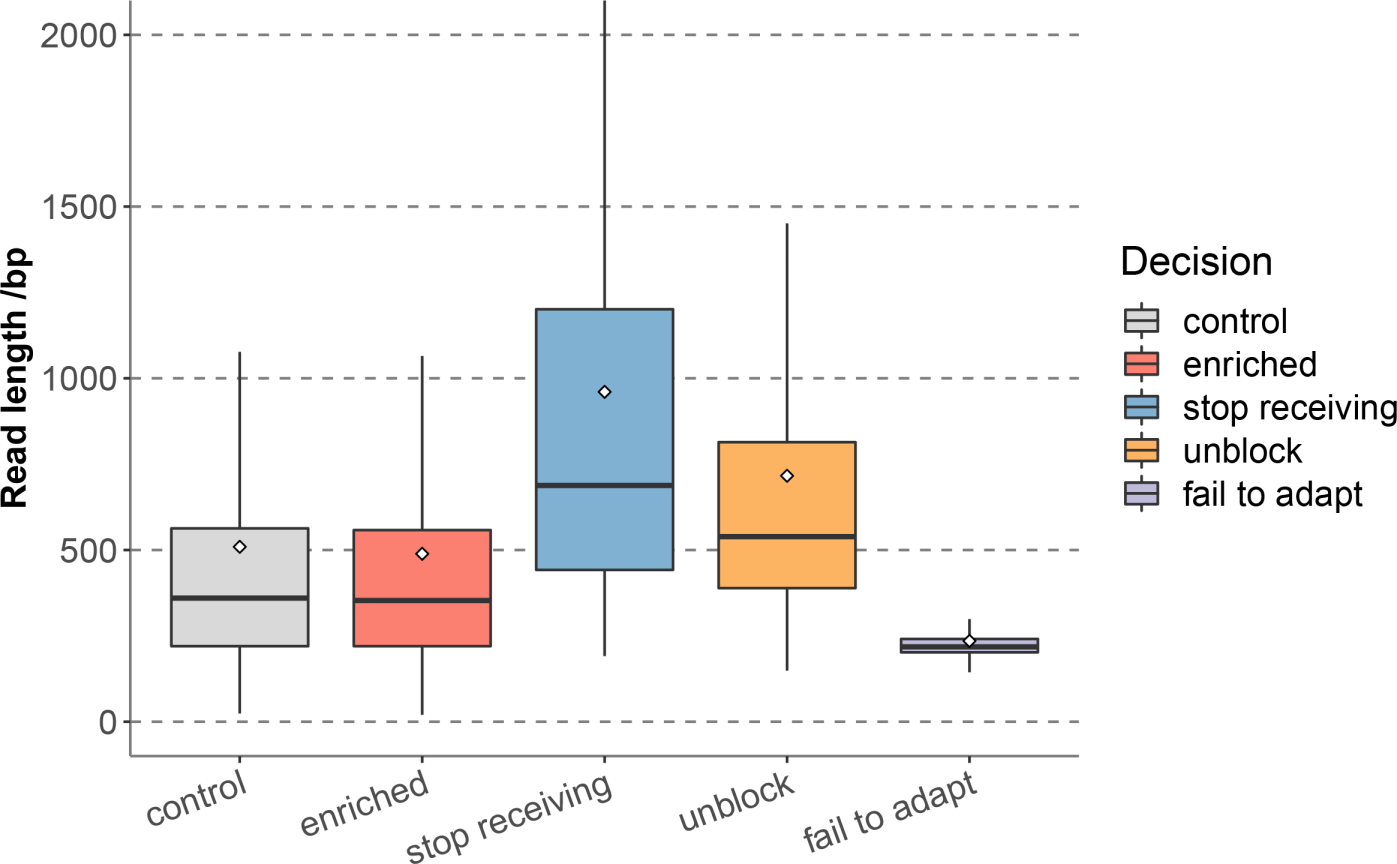
Read length distribution of each sequence groups for nanopore adaptive sequencing.

In 12 hours of sequencing, a total of 2.53 Gb data was obtained, including 5.07 × 10^6^ reads, of which the enriched group generated 1.24 Gb data, corresponding to 2.53 × 10^6^ reads, and the control group generated 1.29 Gb data, corresponding to 2.54 × 10^6^ reads. The enriched group reads were further classified according to different decisions of adaptive sequencing, among which stop receiving reads accounted for 0.03% (649), and unblock reads accounted for 52.69% (1,331,115), indicating that the proportion of non-target reads were much higher than the retained target reads, and the enriched group effectively identified and removed non-target sequences. In addition, the proportions of fail to adapt reads was 47.28% (1,194,353). Statistics of the read length distribution of each group (**Figure 1** and **Supplementary Table 1**) showed that the median read length of the enriched group (353bp) and the control group (359bp) were close (**Supplementary Table 2**). The stop receiving reads of the enriched group had the longest median read length (688bp), followed by unblock reads (539bp), while the median read length of fail to adapt reads (218bp) were significantly shorter than the above two groups.

### Comparison of *C. psittaci* data between enriched and control groups in nanopore adaptive sequencing

**Figure 2:**
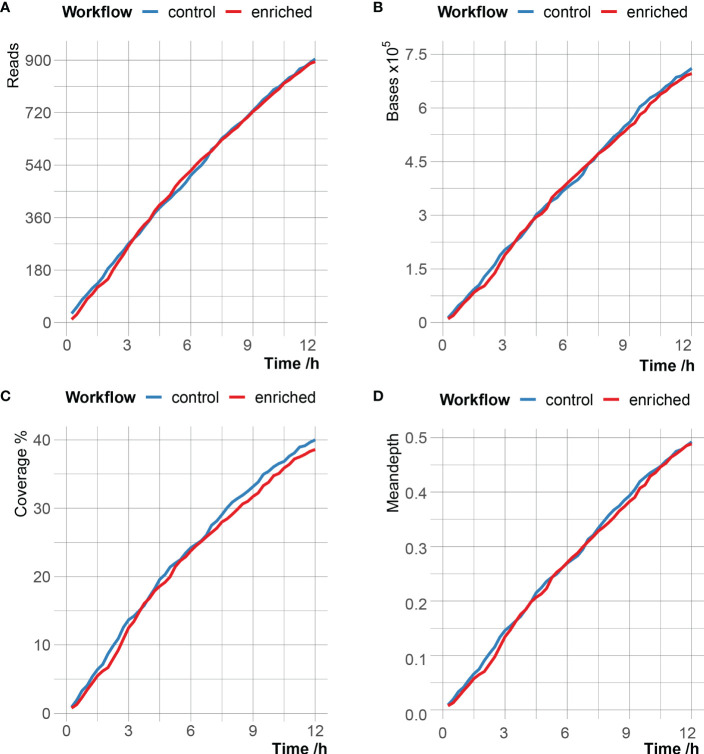
Comparison of *C. psittaci* data in enriched group and control group over time with the number of reads **(A)**, the number of bases **(B)**, the breadth of genome coverage **(C)** and mean depth of genome coverage **(D)** of *C. psittaci*.

According to different time stamps, the *C. psittaci*-specific reads generated in the enriched group and the control group were counted, and the number of reads, bases, genome coverage and mean depth of *C. psittaci* between the two groups were very close (**Figure 2** and **Supplementary Table 3**). After 12 hours of sequencing, the enriched group obtained a total of 895 C*. psittaci* reads, with a cumulative total base of 696kb, corresponding to 38.59% genome coverage and 0.49× genome sequencing depth; the control group produced 904 C*. psittaci* reads, with a cumulative total base of 711kb, corresponding to 40.01% genome coverage and 0.49 × genome sequencing depth (**Supplementary Table 4**). However, of the 895 C*. psittaci* reads in the enriched group, the proportion of the reads from stop receiving group reached 72.4% (648 reads), which was much higher than that in the unblock (2.01%) and fail to adapt (25.59%) groups. Meanwhile, the read proportion matched to *C. psittaci* in the stop receiving group was 99.85%, which was also much higher than that of the unblock (<0.01%) and fail to adapt (0.02%) groups, indicating that adaptive sequencing had enrichment effect on *C. psittaci* reads.

As adaptive sequencing requires at least 1 second to make the judgement, we evaluated whether there was enrichment effect by removing reads shorter than 450bp and found that the matched data of *C. psittaci* was not increased in the enriched group (**Supplemantary Table 5**). The proportion of reads shorter than 450bp in the enriched group (68.52%) and control group (67.93%) were comparable. However, the *C. psittaci* reads belonging to stop receiving tag in the enriched group raised from 72.4% to 97.0%, indicating that adaptive sequencing selected the targeted reads with higher accuracy after removing short reads (**Figure S1**). In addition, the proportion of fail to adapt reads less than 450bp reached 99.01%, indicating that these reads may be too short to be detected by adaptive sampling.

### Comparison of nanopore adaptive sequencing and mNGS

Compared with the mNGS results of the previous study, the proportion of *C. psittaci* reads in the enriched group, the control group and mNGS was similar, accounting for 0.035%, 0.035% and 0.036% of the total sequencing reads, respectively. mNGS provided higher data throughput, with a coverage of 99.51% of the reference genome of *C. psittaci*, and a genome depth of 6.81× (**Table 1**). The total data throughput of the nanopore sequencing enriched group and the control group is only 11.27% and 11.76% compared with that of mNGS. Although both nanopore adaptive sequencing and standard sequencing can be effectively used for the identification of pathogens, the coverage of the *C. psittaci* reference genome is less than 40% and the sequencing depth is basically only 1× for most of the sites (**Figure S2**).

**Table 1:** Comparison of *C. psittaci* data in adaptive sequencing and mNGS.

Group	total bases	total reads	reads of *C. psittaci*	reads propotion of *C. psittaci* (%)	genome coverage *of C. psittaci* (%)	mean depth *of C. psittaci* genome (×)
Enriched*	1238381610	2526117	889	0.035	38.45	0.49
Control*	1292036827	2544516	902	0.035	39.92	0.49
mNGS	10985168800	109851688	39385	0.036	99.51	6.81

*Enriched, enriched group of nanopore sequencing, Control: control group of nanopore sequencing.

## Discussion

In this study, nanopore adaptive sequencing was performed on a bronchoalveolar lavage fluid sample from a patient with *C. psittaci* infection. Combined with the results of previous mNGS, the feasibility of adaptive sequencing in clinical sample pathogen identification was evaluated. Adaptive sequencing identified 72.4% *C. psittaci* reads with high accuracy (99.85%), and ejected 52.69% reads from the sample in real-time. Meanwhile, the proportion of target reads in adaptive sequencing is comparable to that of standard nanopore sequencing and mNGS, which indicates that adaptive sequencing can effectively identify the corresponding target pathogen reads without changing the proportion of species in the sample. Combined with the real-time sequencing advantages of the nanopore sequencing platform, it holds great promise in the rapid identification of pathogens.

Previous studies have found that adaptive sequencing can generate more microbial-related data than standard nanopore sequencing (Ong et al., 2022b), and can achieve a maximum enrichment effect of about 5-fold for mocked microbial community samples (Kovaka et al., 2021; Payne et al., 2021; Martin et al., 2022). In this study, the base yield of *C. psittaci* in the enriched group was comparable of that in the control group, indicating that adaptive sequencing did not significantly increase the effective data yield of the pathogen. This may be related to the short DNA length of the sample. Previous studies have found that the enrichment effect of adaptive sequencing is correlated to the length of the DNA fragment (Martin et al., 2022). In this study, the fragment size of the sample DNA was quality-controlled and the median length was less than 1000bp. However, the speed of the sequences passing through the nanopore was about 450 bases per second (Payne et al., 2021), and the judgment of adaptive sequencing required at least 1 second. Short fragments of the sample make the alignment tool unable to provide timely feedback, resulting in a large number of fail to adapt reads, which account for 47.28% of the total reads. The proportion of reads less than 300 bases in the control group was 42.66%, which is close to the proportion of fail to adapt reads in the enriched group. Meanwhile, ejecting of short fragments and allowing next read sequencing cost longer time than directly sequencing of the fragments, thus reducing the enrichment efficiency. The integrity of the sample DNA is a key factor to obtain better enrichment performance. It’s recommended to select fresh collected samples and avoid freeze-thaw. In addition, high molecular weight DNA extraction methods (Quick, 2019; Boughattas et al., 2021; Petersen et al., 2022; Trigodet et al., 2022) can be used to maintain longer read length, which may help to improve the enrichment effect of adaptive sequencing.

This study has some limitations. Nanopore sequencing is limited by the low throughput and the amount of effective pathogen-related data, which makes it difficult to assemble the whole genome sequence of the pathogen. The adaptive sequencing generated unbiased data along the *C. psittaci* genome but lower coverage due to limited output. To compare with mNGS results, we have used a previous sample, which has been stored for a long time. The partially degraded sample resulted in more short fragments and reduced the efficiency of nanopore adaptive sequencing. In addition, only one sample is included in this study, large-scale research should be carried out to further evaluate the utility of adaptive sequencing in clinical pathogen detection.

## Conclusions

Our Study highlighted that nanopore adaptive sequencing can effectively identify sequences of target pathogen in real-time, but fail to increase the targeted data of pathogens in this case. Further studies need to address how to enrich pathogens with higher data output, which might be a great obstacle for the application of adaptive sequencing in rapid clinical diagnostics of infectious diseases.

## Data availability statement

The original contributions presented in the study are included in the article/**Supplementary Material**. Further inquiries can be directed to the corresponding authors.

## Author contributions

YL, JL, KW performed experiment. YD, SZ, HG and LY performed formal analysis. MN, ZH and LJ collected samples. YL and YD wrote and revised the draft of the manuscript. HL, PL and HS designed the study and revised the manuscript. All authors contributed to the article and approved the submitted version.
