# Supplementary figures and images for: Application of nanopore adaptive sequencing in pathogen detection of a patient with Chlamydia psittaci infection
Source: Front Cell Infect Microbiol. 2023 Jan 23;13:1064317. doi: 10.3389/fcimb.2023.1064317 (PMC9900021; doi:10.3389/fcimb.2023.1064317)

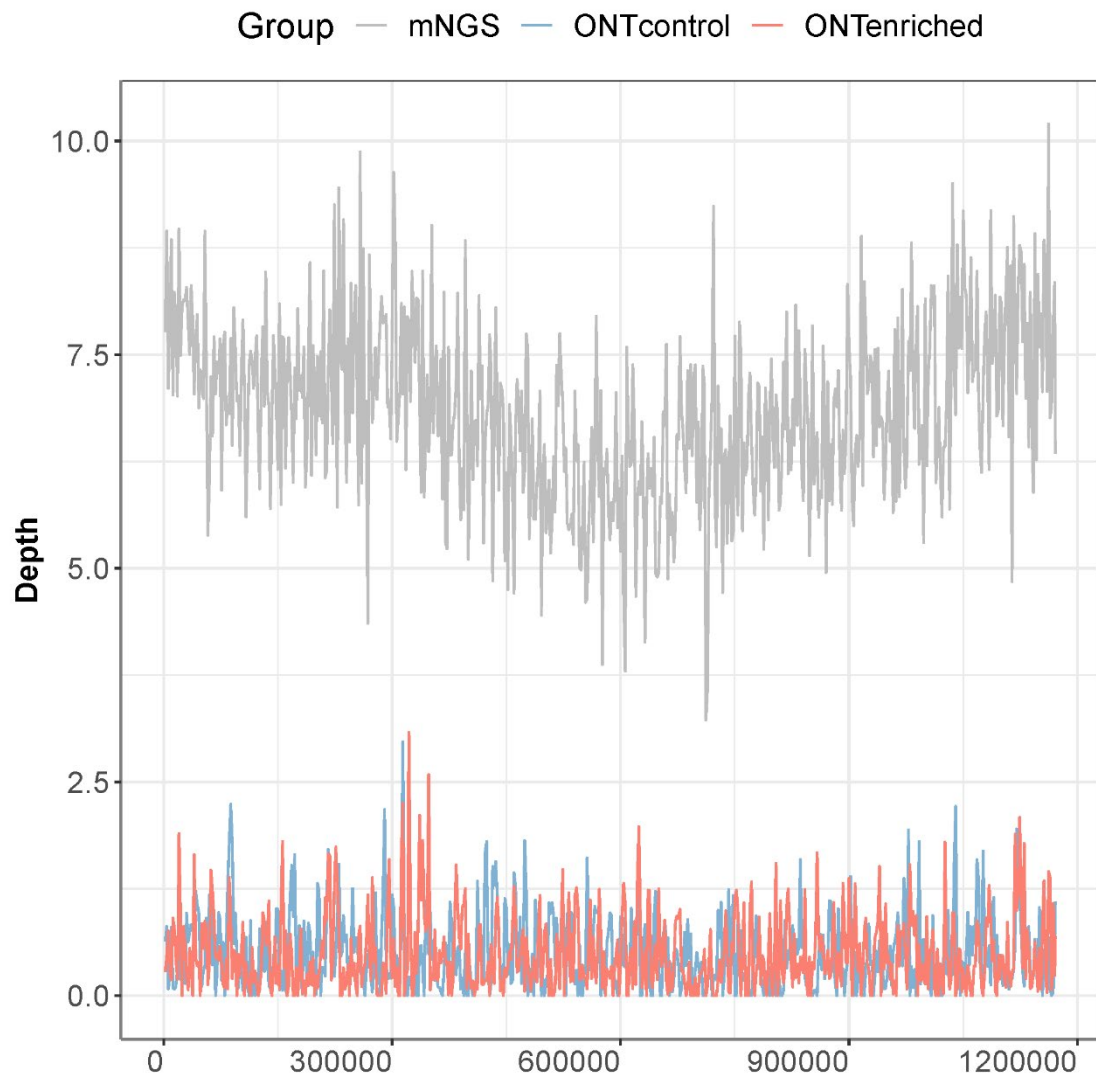

**Fig.S1** Genome depth of *C. psittaci* in mNGS, ONT control and ONT enriched group

Supplement: Supplementary file 1 [file Image_1.pdf]

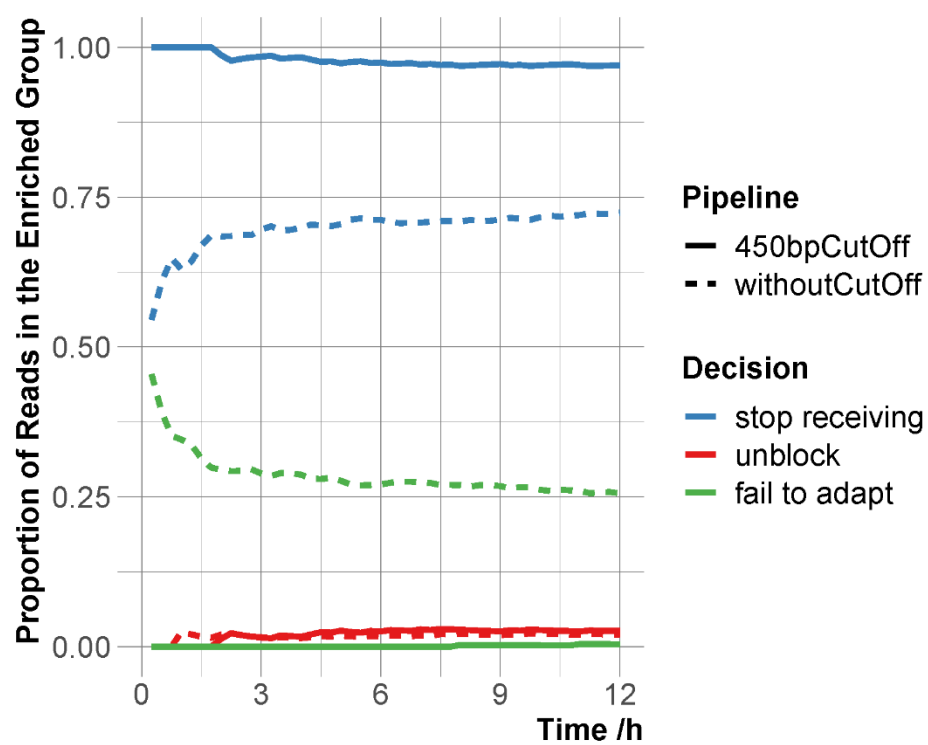

**Fig.S2** Proportion of reads with three decisions in the enriched group

Supplement: Supplementary file 2 [file Image_2.pdf]
