# Supplementary material for: Application of nanopore adaptive sequencing in pathogen detection of a patient with Chlamydia psittaci infection
Source: Front Cell Infect Microbiol. 2023 Jan 23;13:1064317. doi: 10.3389/fcimb.2023.1064317 (PMC9900021; doi:10.3389/fcimb.2023.1064317)
Supplement: Supplementary file 3 [file Table_1.pdf]

Supplementary Table 1. Summary statistics of read length in five groups

Decision	Median length	Mean length	Q1	Q3	IQR
control	360	509	220	563	343
enriched	353	489	220	558	338
fail to adapt	218	235	202	241	39
unblock	539	716	389	814	425
stop receiving	688	960	442	1201	759
