# Supplementary material for: Application of nanopore adaptive sequencing in pathogen detection of a patient with Chlamydia psittaci infection
Source: Front Cell Infect Microbiol. 2023 Jan 23;13:1064317. doi: 10.3389/fcimb.2023.1064317 (PMC9900021; doi:10.3389/fcimb.2023.1064317)
Supplement: Supplementary file 4 [file Table_2.pdf]

Supplementary Table 2. Welch’s t-test of read length between two groups

Decision1	Decision2	Hypothesis test	P value	Effect size estimation	Effect size	Effect size CI
control	enriched	Welch's t-test	<0.0001	Hedge's g	0.039	[0.034, 0.045]
control	stop receiving	Welch's t-test	<0.0001	Hedge's g	0.777	[0.704, 0.85]
control	unblock	Welch's t-test	<0.0001	Hedge's g	0.369	[0.406, 0.406]
control	fail to adapt	Welch's t-test	<0.0001	Hedge's g	0.57	[0.627, 0.627]
stop receiving	unblock	Welch's t-test	<0.0001	Hedge's g	0.47	[0.398, 0.543]
stop receiving	fail to adapt	Welch's t-test	<0.0001	Hedge's g	9.77	[10.747, 10.747]
unblock	fail to adapt	Welch's t-test	<0.0001	Hedge's g	1.265	[1.391, 1.391]
