# Supplementary material for: Application of nanopore adaptive sequencing in pathogen detection of a patient with Chlamydia psittaci infection
Source: Front Cell Infect Microbiol. 2023 Jan 23;13:1064317. doi: 10.3389/fcimb.2023.1064317 (PMC9900021; doi:10.3389/fcimb.2023.1064317)
Supplement: Supplementary file 5 [file Table_3.pdf]

Supplementary Table 3. The results of linear mixed regression model

Variable		Coef	Std.Err.	z	P > |z|	95% CI
Reads	Intercept	34.284	4.481	7.651	0	[25.502, 43.066]
	Time	76.049	0.637	119.424	0	[74.801, 77.297]
	**Group Var**	**0**	1.437			
Bases	Intercept	0.094	0.041	2.289	0.022	[0.014, 0.175]
	Time	0.605	0.004	157.446	0	[0.597, 0.612]
	**Group Var**	**0.002**	0.025			
Coverage	Intercept	0.024	0.006	4.139	0	[0.013, 0.035]
	Time	0.033	0	77.184	0	[0.032, 0.034]
	**Group Var**	**0**	0.005			
Mean depth	Intercept	0.008	0.004	2.302	0.021	[0.001, 0.016]
	Time	0.042	0	143.743	0	[0.042, 0.043]
	**Group Var**	**0**	0.003			
